# Endostar combined with docetaxel or cisplatin in the management of malignant ascites through hyperthermic intraperitoneal chemotherapy: A retrospective cohort study

**DOI:** 10.3892/ol.2026.15463

**Published:** 2026-01-14

**Authors:** Jing Wu, Dashan Yin, Jin Qian, Yi Zhou, Yajun Wu, Ganlu Zhang

**Affiliations:** 1 Department of Oncology, Zhejiang Hospital, Hangzhou, Zhejiang 310030, P.R. China; 2 School of Medicine, Zhejiang University, Hangzhou, Zhejiang 310058, P.R. China; 3 The Second School of Clinical Medical, Zhejiang Chinese Medical University, Hangzhou, Zhejiang 310053, P.R. China; 4 Department of Traditional Chinese Medicine Pharmacy, Zhejiang Hospital, Hangzhou, Zhejiang 310030, P.R. China

**Keywords:** peritoneal metastasis, malignant ascites, Endostar, docetaxel, cisplatin, hyperthermic intraperitoneal chemotherapy

## Abstract

Peritoneal metastases (PM) are a prevalent and treatment-resistant form of recurrence in patients with abdominal tumors. The present study retrospectively examined the clinical performance and potential adverse effects of combining Endostar with docetaxel/cisplatin chemotherapeutic hyperthermic intraperitoneal chemotherapy (HIPEC) for treating malignant ascites (MA). Subjects diagnosed with malignancies confirmed by ascites cell block results and clinical presentations were included within the study, constituting a total of 48 MA cases. The docetaxel group (n=25) was treated with Endostar combined with docetaxel for two cycles. The cisplatin group (n=23) was treated with Endostar combined with cisplatin for two cycles. The objective response rate (ORR; defined as CR + PR), disease control rate (DCR; defined as CR + PR + SD), ascites control time, improvement rate of the Karnofsky Performance Status (KPS) and incidence of major adverse reactions were statistically analyzed. There were no significant differences in the ORR (64.0 vs. 56.5%), DCR (92.0 vs. 91.3%), improvement rate of KPS (48.0 vs. 43.5%) or ascites control time (55 vs. 46 days) between the docetaxel and cisplatin groups (all P>0.05). Renal function impairment developed in 7 patients (30.4%) in the cisplatin group compared with 1 patient (4.0%) in the docetaxel group (P<0.05). Except for renal injury and blood pressure, there were no statistically significant differences in the incidences of other adverse reactions or treatment-related deaths between the two groups. Overall, the combined application of Endostar and docetaxel/cisplatin HIPEC yielded promising clinical outcomes in the treatment of MA, as indicated by high ORRs and DCRs, significantly improving the quality of life for affected individuals. However, it is worth noting that the incidence of nephrotoxicity was higher in the cisplatin treatment group compared with that in the docetaxel treatment group. Therefore, clinicians should be cautious about nephrotoxicity when administering cisplatin in high-risk patients with PM.

## Introduction

Peritoneal metastases (PM), typically originating from colorectal cancer (1), gastric cancer (2,3), ovarian cancer (4), pancreatic cancer (5) and other abdominal tumors, are typically regarded as the terminal stage of cancer (6). Uncontrolled malignant ascites (MA) is the main complication of PM, leading to abdominal distension, abdominal pain, dyspnea, loss of appetite, nausea, vomiting and decreased mobility, which seriously affects quality of life (7). The effectiveness of existing treatment methods for PM, including extended frequent high-volume puncture drainage, peritoneal venous shunt, diuretics, systemic chemotherapy, intraperitoneal (IP) chemotherapy and immunotherapy, remains limited and does not meet the needs of patients. Given the significantly higher incidence of renal injury observed in the cisplatin group in the present study, clinicians should be cautious about nephrotoxicity when considering this agent for high-risk patients with PM.

Hyperthermic IP chemotherapy (HIPEC) is a specialized treatment method that targets and directly eliminates malignant cells within the abdominal cavity by circulating heated chemotherapy drugs within the peritoneal cavity, especially in patients who have experienced systemic therapy failure (8,9). This technique aims to eliminate free cancer cells, fibrin and other cellular debris by flushing these substances out of the body, which can reduce adhesion. Additionally, HIPEC can enhance the absorption and sensitivity of tumor cells to chemotherapy drugs, increasing the penetration of chemotherapy at the peritoneal surface, resulting in efficient tumor cell death for advanced peritoneal cavity cancers (10). In 1980, Spratt first reported (11) the use of HIPEC specifically for the treatment of abdominal and pelvic malignant tumors, and residual tumors, and this technique has proven to be a promising treatment modality (12). HIPEC is used to treat gastrointestinal cancer, ovarian cancer, pseudomyxoma peritonei and other peritoneal cancer types (13,14). However, this technology is currently underdeveloped, and there are no clear guidelines for drug selection.

Endostar is a vascular endothelial growth factor (VEGF) inhibitor that has been shown to significantly reduce visible ascites formation and tumor burden (15). VEGF overexpression is commonly observed in malignant cancer metastases affecting the abdominal region (16). Prospective multicenter clinical studies have confirmed the efficacy of Endostar and cisplatin injections for controlling malignant pleural effusions (17,18). In recent years, docetaxel has also been reported to control MA (19). In addition, hyperthermia is known to enhance tumor perfusion and increase drug penetration after IP delivery (20).

The present study retrospectively assessed the effectiveness and adverse effects of HIPEC using docetaxel/cisplatin, combined with IP Endostar injections, for treating MA in patients who had failed at least two rounds of systemic chemotherapy regimens. The findings provide valuable insights for improving and developing new therapies for this condition.

## Materials and methods

### Study design and patients

The present retrospective study involved patients treated between July 2019 and December 2020 at the Sandun Campus of Zhejiang Hospital (Hangzhou, China). The eligibility criteria were as follows: i) Patients must have been diagnosed with a malignant tumor, with evidence of cytology or tumor cells in an ascites cell block; ii) patients must have previously undergone systemic chemotherapy of second line or higher, and not undergone a HIPEC procedure in the past 6 months; iii) patients must not have received docetaxel, cisplatin or Endostar before receiving the protocol under investigation; iv) hemoglobin level must be ≥90 g/l (normal range, 120–160 g/l for women and 130–170 g/l for men); v) absolute neutrophil count must be ≥1.5×10^9^/l (normal range, 1.8–7.7×10^9^/l); vi) white blood cell count must be >3.5×10^9^/l (normal range, 4.0–10.0×10^9^/l); vii) platelet count must be ≥85×10^9^/l (normal range, 150–400×10^9^/l); viii) total bilirubin must be ≤1.5 times the upper limit of normal (ULN) (normal range, 3.4–20.5 µmol/l or 0.2–1.2 mg/dl); ix) aspartate aminotransferase and alanine aminotransferase must both be ≤2.5 times the ULN (normal range for AST, 8–40 U/l; normal range for ALT, 7–56 U/l); x) patients must have an expected survival time of >3 months; and xi) patients should have failed at least two rounds of systemic chemotherapy regimens. The exclusion criteria were: i) Uncontrolled central nervous system metastases with manifestations of intracranial hypertension; ii) bleeding tendency, especially marked gastrointestinal bleeding within the past 4 weeks, or currently undergoing thrombolytic or anticoagulant therapy; iii) prior IP infusion of docetaxel, cisplatin or Endostar within 6 months, or concurrent participation in other clinical studies; iv) myocardial infarction within the past 6 months, or current unstable angina pectoris or cardiac insufficiency; v) severe chronic obstructive pulmonary disease and/or respiratory failure, or severe intestinal adhesions; vi) currently uncontrolled severe infection; vii) known allergy to the investigational drug(s) or their excipients; viii) fertile patients unwilling to adopt contraception, and pregnant or lactating women; ix) poor compliance or diagnosed with significant psychiatric disorders causing lack of self-control.

All patients provided written informed consent prior to catheterization and underwent HIPEC via abdominal puncture as part of routine clinical treatment for malignant ascites. This study was approved by the Scientific Research Board of Zhejiang Hospital, who waived the requirement for informed patient consent for study participation.

### Treatment procedure

**Figure 1.**
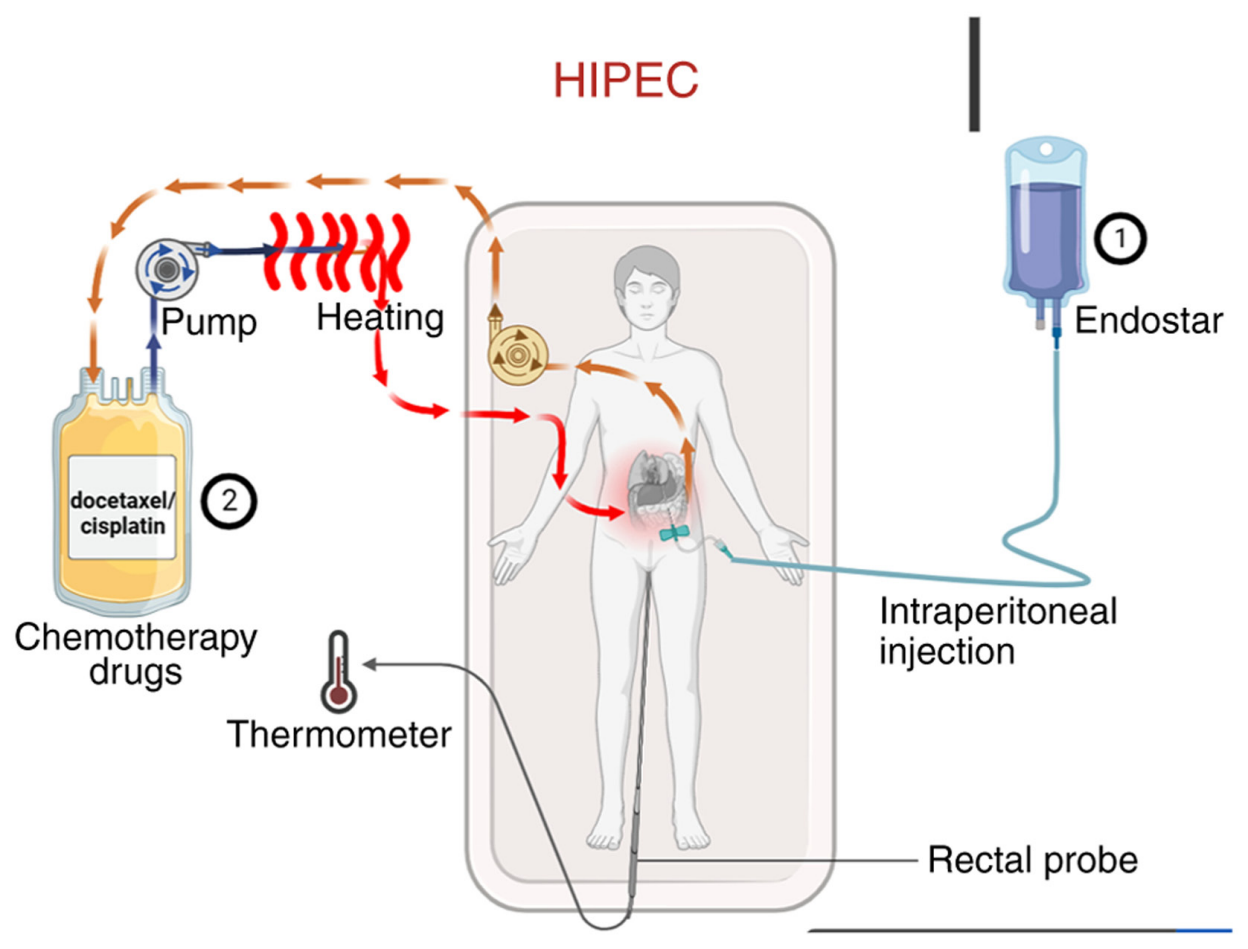
Schematic diagram of docetaxel/cisplatin HIPEC combined with IP Endostar. Point 1: Endostar was injected intraperitoneally. Point 2: Docetaxel or cisplatin was heated and circulated. A thermometer was placed in the rectum to monitor the patient's temperature. IP, intraperitoneal; HIPEC, hyperthermic IP chemotherapy.

Abdominal circumference was measured in all patients before and after treatment. The treatment protocol is outlined in Fig. 1. First, the volume of ascites was assessed via abdominal ultrasonography. Under ultrasound guidance, a single-lumen central venous catheter or an external drainage catheter (6F or 8F) was percutaneously inserted into the abdominal cavity for continuous drainage. Ascites was drained slowly over 1–3 days with the flow regulated not to exceed 1,000 ml/h to prevent complications. Endostar was administered by intraperitoneal injection on days 1, 4 and 7 of the treatment cycle. On day 4, HIPEC was performed immediately following the Endostar injection. The chemotherapeutic agent (docetaxel or cisplatin) was prepared in a perfusion solution, which was then circulated through a closed-loop system equipped with a pump and a heating module. The solution was heated to the target therapeutic temperature (42–43°C) and infused into the abdominal cavity; it was maintained in circulation for the prescribed duration to allow for continuous hyperthermic perfusion of the peritoneal surface, before being returned to the system for reheating and recirculation. Core body temperature was monitored in real-time throughout the HIPEC procedure using a rectal temperature probe. Ascites drainage statistics were collected. HIPEC was administered using a thermochemotherapy perfusion device (RHL-2000A; Jilin Maida Medical Device Co., Ltd.) with careful temperature control of the body between 43 and 45°C. Prior to chemotherapeutic drug infusion, the abdominal cavity was effectively rinsed with 1,000-2,000 ml of warm normal saline.

All patients received IP injections of Endostar (60 mg) on days 1, 4 and 7 of a 21-day cycle, for a total of two cycles. The Endostar timing was decided based on the vascular normalization window principle, as recombinant human endostatin can temporarily normalize the structure and function of the tumor vasculature, with this optimal window typically occurring between 3 to 5 days after administration. Therefore, the concurrent IP administration of Endostar and HIPEC chemotherapeutic agents on day 4 was designed to leverage this transient window to reduce interstitial pressure and promote the deep and uniform penetration of chemotherapeutic drugs into the tumor tissues, achieving spatiotemporal synergism. This regimen aligns with the recommendations of the Expert Consensus on the Clinical Application of Recombinant Human Endostatin for the Treatment of Malignant Serous Cavity Effusions (21) and has been validated as effective in multiple clinical studies (22–25). As well as Endostar, 25 patients in the docetaxel group received 60 mg/m^2^ docetaxel intraperitoneally on day 4 as circulating hyperthermic perfusion chemotherapy, with 21 days per cycle, for a total of two cycles. Oral administration of 4 mg dexamethasone tablets was started the day before docetaxel treatment, for a total of 3 days. In the cisplatin group, 23 patients received 60 mg/m^2^ cisplatin on day 4 as IP circulating hyperthermic perfusion chemotherapy, with 21 days per cycle, for a total of two cycles. During the course of treatment, diuresis and albumin supplementation were administered as supportive treatments according to specific conditions, routine blood tests were performed, liver and kidney functions were monitored, and adverse reactions were observed.

The selection of the treatment approach was not randomized. Within Zhejiang Hospital, patients with gastric, ovarian and pancreatic cancer are recommended either cisplatin or docetaxel treatment, while for patients with colon cancer, only cisplatin is used. These recommendations are based on clinical practice guidelines and individual patient characteristics. Moreover, in line with standard clinical practice to avoid cisplatin-associated nephrotoxicity and neurotoxicity, patients with renal insufficiency (eGFR <60 ml/min) or neuropathy received docetaxel rather than cisplatin.

### Outcomes

The ascites control time was defined as the duration from achieving a complete response (CR) or partial response (PR) until the first documented evidence of progressive disease (PD) and was calculated from the end of the second cycle of treatment. The abdominal circumference was measured twice a week, and follow-up examinations were conducted at the hospital 4 weeks after the end of treatment, followed by collection of abdominal circumference measurements by telephone. The World Health Organization evaluation standard (26) classifies ascites that has completely subsided for >4 weeks as a CR, ascites that has decreased by >50% and been maintained for >4 weeks as a PR, ascites that has decreased by <50% or increased by <25% and been maintained for >4 weeks as SD, and ascites that has increased by >25% as PD. Objective response rate (ORR)=(CR + PR)/total number of cases ×100. Disease control rate (DCR)=(CR + PR + SD)/total number of cases ×100. If the ascites reached PD, follow-up was terminated after 90 days. During the follow-up period, there were no deaths or patients lost to follow-up.

The changes in Karnofsky Performance Status (KPS) score before and after treatment were evaluated according to the Karnofsky scoring standard (27). After treatment, a KPS increase of ≥10 points was evaluated as an improvement in quality of life (QOL), a change of <10 points was evaluated as stable and a decrease of ≥10 points was evaluated as decreased QOL (28).

According to the National Cancer Institute Common Toxic Reaction Standard CTC V4.0, the toxicity grading standard is divided into grades 1–5, of which grades 1–2 are low-grade reactions, grades 3–4 are severe reactions and grade 5 indicates death (29).

### Follow-up

The follow-up protocol was defined as follows: Once PD was reached, the endpoint was determined. Further intensive follow-up was therefore unnecessary for scientific reasons, and also to reduce the burden on patients. Follow-up was terminated 90 days after PD, or earlier if the patient died or was lost to follow-up, while patients without PD were followed until death, loss to follow-up or study end.

### Statistical analysis

Statistical software SPSS (version 26.0; IBM Corp.) was used for the data analysis. The ascites control time was defined as the duration from achieving a CR or PR until the first documented evidence of PD and was analyzed using the Kaplan-Meier method and differences between groups were compared using the log-rank test. Normally distributed continuous variables (as assessed using quantile-quantile plots) are expressed as the mean ± standard deviation and compared using an unpaired t-test, while non-normally distributed variables are presented as the median (interquartile range) and compared using the Wilcoxon rank-sum test. Count data were compared between the groups using the χ^2^ test or Fisher's exact probability method. P<0.05 is considered to indicate a statistically significant difference.

## Results

### Patient's basic characteristics

**Figure 2.**
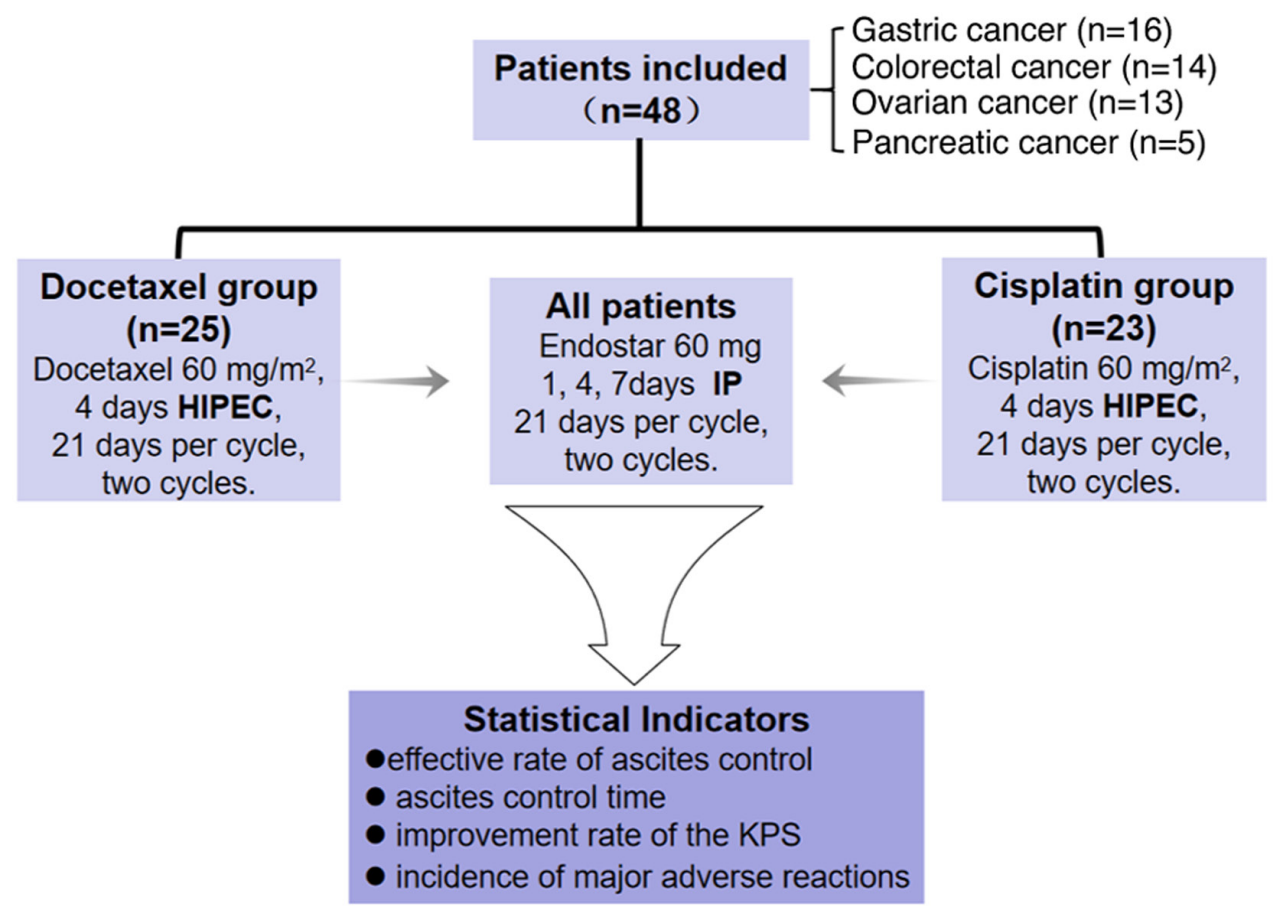
Process scheme of the study. A total of 48 patients were included for the final analysis, including 16 patients with gastric cancer, 14 patients with colorectal cancer, 13 patients with ovarian cancer and 5 patients with pancreatic cancer. IP, intraperitoneal; HIPEC, hyperthermic IP chemotherapy; KPS, Karnofsky Performance Status.

As illustrated in Fig. 2, a collective total of 48 patients were included in this research, with 16 patients diagnosed with gastric cancer, 14 patients diagnosed with colorectal cancer, 13 patients diagnosed with ovarian cancer and 5 patients diagnosed with pancreatic cancer. Comparing the cisplatin and docetaxel groups, the age (55.9±15.4 vs. 55.5±13.8 years; t=0.350; P=0.927), sex [male: 11 (47.8%) vs. 10 (40.0%); χ^2^=0.298; P=0.585], primary tumor type (the most common type was gastric cancer: 8 (34.8%) vs. 8 (32.0%); χ^2^=0.480; P=0.923), KPS score [≥60 points: 14 (60.9%) vs. 16 (64.0%); χ^2^=0.050; P=0.823], ascites volume [<3,000 ml: 10 (43.5%) vs. 9 (36.0%); χ^2^=0.280; P=0.597] and previous treatment option [second-line: 9 (39.1%) vs. 8 (32.0%); χ^2^=0.266; P=0.606] of the two groups were evenly distributed and the results were comparable (Table I).

### Objective efficacy evaluation

In Table II, among the participants in the docetaxel group, 3 patients achieved a CR, 13 achieved a PR, 7 were stable and 2 exhibited PD, with an ORR of 64.0% and a DCR of 92.0%. In the cisplatin group, 2 patients achieved a CR, 11 achieved a PR, 8 were stable and 2 exhibited PD, with an ORR of 56.5% and a DCR of 91.3%. There were no statistically significant differences between the two groups in terms of either ORR (χ^2^=0.280, P=0.597) or DCR (P>0.999, Fisher's exact test).

### QOL of patients

KPS scores were utilized to gauge the QOL. As shown in Table III, KPS improvement rates were 48.0 and 43.5% for the docetaxel and cisplatin groups, respectively. There was no significant difference in the QOL between the two groups (χ^2^*=*0.967; P*=*0.617).

**Figure 3.**
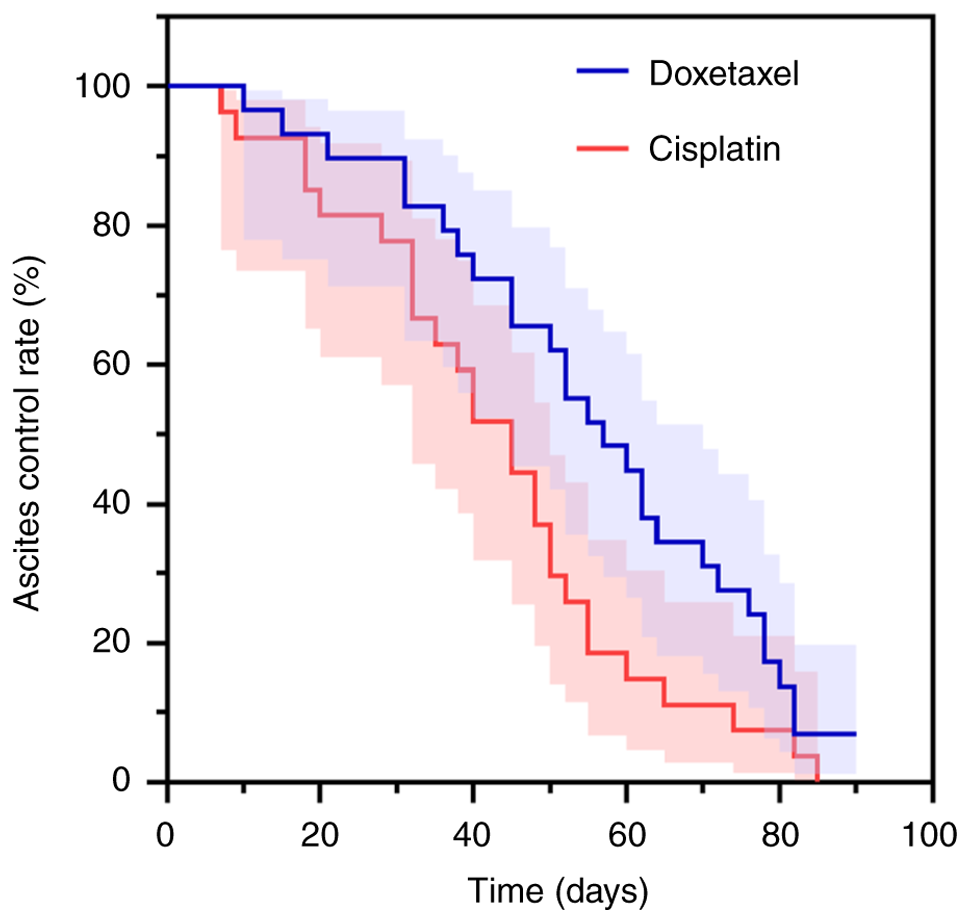
Comparison of ascites control between the two groups of patients.

The median ascites control time was 55 days (95% CI: 46.892–63.108) in the docetaxel group and 46 days (95% CI: 34.261–57.739) in the cisplatin group. When comparing the control time between the two groups, the difference was not statistically significant (log-rank χ^2^*=*0.934; P*=*0.334) (Fig. 3).

### Security analysis

There were no significant differences between the two groups in terms of the incidence of leukopenia (χ*^2^*=0.680; P=0.712), anemia (χ*^2^*=0.473; P=0.789), thrombocytopenia (χ*^2^*=0.762; P=0.683), nausea and vomiting (χ*^2^*=1.451; P=0.484), anorexia (χ*^2^*=0.309; P=0.857), fatigue (χ*^2^*=0.071; P=0.965), constipation (χ*^2^*=0.371; P=0.573), abdominal pain and diarrhea (χ*^2^*=0.285; P=0.867), hepatic damage (χ*^2^*=0.084; P=0.772), or palpitation/chest tightness (χ*^2^*=0.084; P=0.772). However, the incidence of kidney damage was significantly higher in the cisplatin group than in the docetaxel group (χ*^2^*=6.194; P=0.045). Additionally, there was a significant difference between the two groups with regard to the elevation of blood pressure (χ*^2^*=5.135; P=0.023) (Table IV).

## Discussion

Advanced tumor PM can result in the development of MA, which significantly impairs patient survival and QOL (30). The development of MA is a multifaceted and intricate physiological process that is intricately linked to the impediment of lymphatic drainage, tumor angiogenesis and alterations in microvascular permeability (31). VEGF stimulates tumor cells and mesothelial cells, causing vascular growth factors such as TNF-α, TGF-β, VEGF and IL-8 to increase in malignant effusions (32). The VEGF level of MA is significantly higher than that of benign ascites and VEGF levels are correlated with a worse prognosis (33). The ‘peritoneal-plasma barrier’ restricts macromolecular drug absorption via the peritoneum, which enables a high drug concentration in the abdominal cavity while maintaining low peripheral blood drug levels (34). HIPEC, a recent therapeutic strategy, promotes deep tissue penetration of chemotherapeutic drugs, enhancing their concentration in tumor cells, and achieving a positive therapeutic effect.

**Figure 4.**
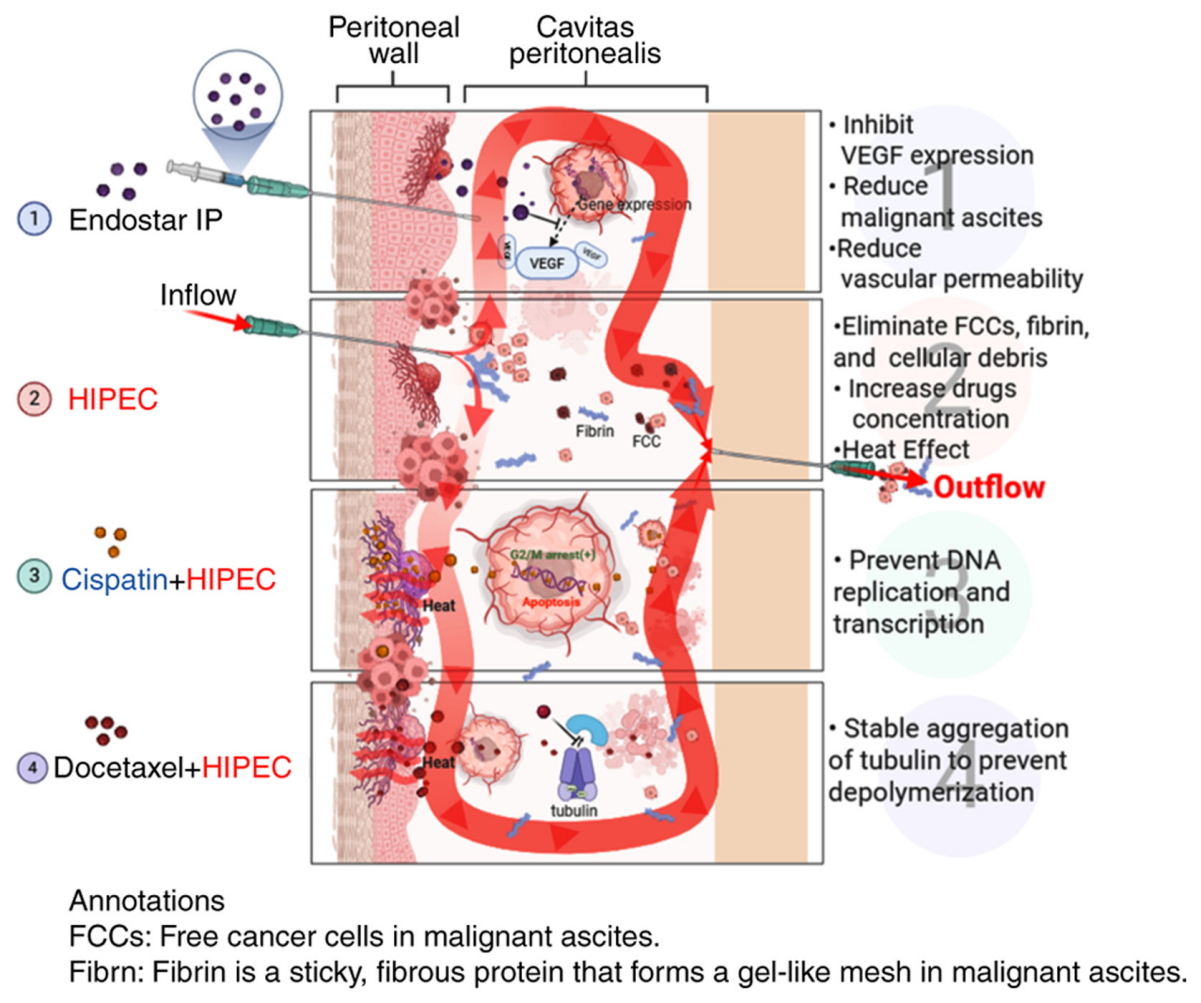
Mechanisms of docetaxel/cisplatin HIPEC combined with IP Endostar in the treatment of MA. As shown in point 1, the IP Endostar injection inhibits VEGF expression, reduces MA and reduces vascular permeability. A shown in point 2, when HIPEC is performed, drug concentration and temperature increase. As shown in points 3 and 4, the addition of cisplatin can prevent DNA replication and transcription, while the addition of docetaxel can stabilize the aggregation of tubulin and prevent depolymerization. MA, malignant ascites; IP, intraperitoneal; HIPEC, hyperthermic IP chemotherapy; FCC, free cancer cell. Created in BioRender, https://app.biorender.com/illustrations/64297f7a20eede8ed30124a8?slideId=3d6d849d-02c8-4b18-b5ee-18396b8d452e.

As shown in Fig. 4, the general mechanism of Endostar combined with docetaxel/cisplatin HIPEC in the treatment of MA may be as follows: i) Endostar inhibits vascular endothelium proliferation, differentiation and migration, reducing blood vessel filtration area and permeability, reducing material exudation and controlling MA osmotic pressure, thereby reducing effusion (7,35,36). ii) HIPEC treatment kills MA tumor cells by repeated washing, causing anoikis and tumor cell detachment. Heat changes the tumor cell membrane and vascular permeability, reducing drug metabolism and increasing drug concentration (37,38). Heat shock protein activation by heat can induce an autoimmune attack on tumor cells, block angiogenesis and cause protein denaturation (39). iii) Cisplatin can directly enter the tumor cell nucleus to prevent DNA replication and transcription to achieve the purpose of killing tumor cells (40). iv) Docetaxel binds to tubulin subunits after entering the body, stably accumulates tubulin, prevents depolymerization and inhibits tumor cell proliferation (41).

Cisplatin and Endostar are both commonly used drugs for HIPEC and have various indications for use. Cisplatin is conventionally indicated for peritoneal malignancies, particularly ovarian cancer (42), whereas Endostar is commonly employed in the management of malignant ascites (43). Zhao *et al* (35) reported that the disease control rate was 87.0% for pleural effusion and ascites using Endostar combined with cisplatin, while Fu *et al* (44) showed that patients who received HIPEC with cisplatin plus docetaxel had a longer median overall survival time compared with those who received cisplatin plus mitomycin. A previous study revealed that detectable cisplatin concentrations persisted for at least 6 h post-HIPEC (45), while Endostar was proven effective for reducing the expression of VEGF and other factors, including fibroblast growth factor-2, transforming growth factor-β1, and platelet-derived growth factor-B (46). However, the major side effects of cisplatin include acute and chronic nephrotoxicity, both systemic and IP. Hakeam *et al* (47) reported that 3.7% of patients experienced acute kidney injury after HIPEC with cisplatin, while Gómez-Ruiz *et al* (48) showed that 7.2% of patients developed acute renal dysfunction after HIPEC. Cisplatin can induce early proximal tubular injury, leading to acute or subacute tubular necrosis. In addition, cisplatin can cause a gradual and irreversible decline in the long-term filtration capacity of the glomerulus, leading to chronic renal failure. This toxicity is the main reason for limiting the IP injection of cisplatin (37). Previous studies showed that the incidence of acute renal failure after HIPEC combined with cisplatin was from 1.3 to 40.4%, and 8.5% developed into grade 3–4 kidney injury; moreover, these acute or chronic renal failures contributed to 4.3% of long-term dialysis patients (49,50), which seriously affected subsequent consolidation therapy with other anticancer drugs or further treatment after recurrence. In the present study, the incidence of renal impairment in the cisplatin group was 30.4%, which is similar to that reported in the aforementioned previous studies. Docetaxel is a semi-synthetic taxoid drug that is widely used to treat non-small cell lung, breast, gastric and ovarian cancer (51). Studies have shown that the area under the curve (AUC) of peritoneal injection is nearly 1,000 times higher than that of intravenous injection, and the peak concentration in the peritoneum is ~200 times higher than that in the plasma, making docetaxel a suitable drug for HIPEC application (52,53). The present study retrospectively evaluated the efficacy and safety of docetaxel/cisplatin combined with Endostar for the treatment of MA. The objective remission rate (64.0 vs. 56.5%), DCR (92.0 vs. 91.3%), improvement in the KPS score (48 vs. 43.5%) and median control time of ascites (55 days vs. 46 days) in the two groups were similar. However, in terms of the occurrence of adverse reactions, the cisplatin group had higher renal toxicity; 5 patients had grade 1–2 renal damage, and after symptomatic treatments such as diuresis and kidney protection (such as reduced glutathione Bailing capsule use), the renal function recovered to normal. Overall, 2 patients had grade 3–4 renal damage, and 1 patient developed chronic renal failure and received hemodialysis maintenance treatment. The incidence of renal impairment was much lower in the docetaxel group than in the cisplatin group, and the underlying reason could be associated with the different metabolism of the two drugs. Miller *et al* (54) reported that cisplatin is primarily excreted through the kidneys and may lead to the damage and necrosis of renal tubular epithelial cells (54). Meanwhile, docetaxel is primarily metabolized in the liver by the cytochrome P450 enzyme system and has low direct toxicity to the kidneys (55). A more direct comparison was shown in the study performed by Kurokawa *et al* (56), where increased creatinine levels were only observed in patients receiving cisplatin plus S-1, while patients with docetaxel plus S-1 exhibited normal creatinine levels (56).

Other adverse effects were similar between the two groups in the present study, and there was no significant difference in efficacy. In comparison to cisplatin, the treatment of MA with docetaxel combined with HIPEC can improve patients' QOL, induce minimal adverse effects on renal function and display fewer adverse reactions. Notably, psychological and social factors are also important for patients' QOL, while the KPS score used in the present study focuses more on physical function. In future studies, the Medical Outcomes Study Social Support Survey (57), Symptom Checklist 90 (58) and other tools should also be evaluated.

The present study had several limitations. Firstly, the sample size was relatively small, with only 48 participants included in the final analysis. This limited number resulted in insufficient statistical power, which may have reduced the ability to detect true differences in ORR, improvement of KPS scores and ascites control time between the two groups. Furthermore, although an attempt was made to perform multivariable analyses to adjust for potential confounding factors, these models failed to converge due to data sparsity issues, which was a direct consequence of the small sample size, therefore yielding no reliable results. Consequently, these findings require rigorous examination in future large-scale cohorts. Secondly, key potential confounders such as the peritoneal cancer index, baseline renal function and specific details of personalized treatment plans were not available for inclusion in the analysis. Thirdly, the retrospective nature of the study means that treatment methods were non-randomly determined based on established guidelines, patients' medical history and current clinical status; therefore, the results remain susceptible to indication bias, emphasizing the necessity for future randomized controlled trials to further validate the findings. Finally, the study relied solely on the KPS score for QOL evaluation. Future studies should employ more comprehensive, multidimensional QOL assessment tools to better capture the full spectrum of patients' psychological and social wellbeing.

In conclusion, the present retrospective study revealed that HIPEC with docetaxel and Endostar exhibited comparable efficacy to a cisplatin-based regimen for controlling MA, but with a significantly more favorable safety profile, particularly with a lower incidence of nephrotoxicity. This indicates that docetaxel may be a preferable agent for MA; however, the finding warrants investigation in future prospective trials.

## Figures and Tables

**Table I.** Comparison of general conditions between the docetaxel (n=25) and cisplatin (n=23) groups.

Variables	Docetaxel group	Cisplatin group	χ^2^/t	P-value
Sex, n (%)			0.298	0.585
Male	10 (40.0)	11 (47.8)		
Female	15 (60.0)	12 (52.2)		
Age, years			0.350	0.927
Average age	55.5±13.8	55.9±15.4		
Age distribution	28-78	30-78		
KPS, n (%)			0.050	0.823
≥60	16 (64.0)	14 (60.9)		
<60	9 (36.0)	9 (39.1)		
Ascites volume, n (%)			0.280	0.597
<3,000 ml	9 (36.0)	10 (43.5)		
≥3,000 ml	16 (64.0)	13 (56.5)		
Prior treatment, n (%)			0.266	0.606
Second-line	8 (32.0)	9 (39.1)		
>Second-line	17 (68.0)	14 (60.9)		
Primary tumor, n (%)			0.480	0.923
Gastric cancer	8 (32.0)	8 (34.8)		
Colorectal cancer	8 (32.0)	6 (26.1)		
Ovarian cancer	6 (24.0)	7 (30.4)		
Pancreatic cancer	3 (12.0)	2 (8.7)		

KPS, Karnofsky Performance Status.

**Table II.** Comparison of curative effects between the docetaxel (n=25) and cisplatin (n=23) groups.

Response	Docetaxel group	Cisplatin group	χ^2^	P-value
CR, n (%)	3 (12.0)	2 (8.7)		
PR, n (%)	13 (52.0)	11 (47.8)		
SD, n (%)	7 (28.0)	8 (34.8)		
PD, n (%)	2 (8.0)	2 (8.7)		
ORR, n (%)	16 (64.0)	13 (56.5)	0.280	0.597
DCR, n (%)	23 (92.0)	21 (91.3)	-	>0.999^a^

a Calculated using Fisher's exact test due to small expected frequencies. CR, complete response; PR, partial response; SD, stable disease; PD, progressive disease; ORR, objective response rate; DCR, disease control rate.

**Table III.** Improvement of quality of life between the docetaxel (n=25) and cisplatin (n=23) groups.

Status	Docetaxel group	Cisplatin group	χ^2^	P-value
Improvement, n (%)	12 (48.0)	10 (43.5)		
Stabilization, n (%)	11 (44.0)	9 (39.1)		
Declined, n (%)	2 (8.0)	4 (17.4)	0.967	0.617

**Table IV.** Adverse reactions between the docetaxel (n=25) and cisplatin (n=23) groups.

Variables	Docetaxel group	Cisplatin group	χ^2^	P-value
Leukopenia, n (%)				
I–II	6 (24.0)	4 (17.4)		
III–IV	2 (8.0)	1 (4.3)	0.680	0.712
Anemia, n (%)				
I–II	5 (20.0)	4 (17.4)		
III–IV	1 (4.0)	2 (8.7)	0.473	0.789
Thrombopenia, n (%)				
I–II	4 (16.0)	6 (26.1)		
III–IV	1 (4.0)	2 (8.7)	0.762	0.683
Nausea and vomiting, n (%)				
I–II	4 (16.0)	5 (21.7)		
III–IV	0 (0.0)	1 (4.3)	1.451	0.484
Anorexia, n (%)				
I–II	8 (32.0)	9 (39.1)		
III–IV	2 (8.0)	2 (8.7)	0.309	0.857
Fatigue, n (%)				
I–II	14 (56.0)	12 (52.2)		
III–IV	2 (8.0)	1 (4.3)	0.071	0.965
Constipation, n (%)				
I–II	6 (24.0)	4 (17.4)		
III–IV	0 (0.0)	0 (0.0)	0.371	0.573
Abdominal pain and diarrhea, n (%)				
I–II	4 (16.0)	5 (21.7)		
III–IV	2 (8.0)	2 (8.7)	0.285	0.867
Hepatic damage, n (%)				
I–II	4 (16.0)	3 (13.0)		
III–IV	0 (0.0)	0 (0.0)	0.084	0.772
Kidney damage, n (%)				
I–II	1 (4.0)	5 (21.7)		
III–IV	0 (0.0)	2 (8.7)	6.194	0.045
Palpitation/chest tightness, n (%)				
I–II	4 (16.0)	3 (13.0)		
III–IV	0 (0.0)	0 (0.0)	0.084	0.772
Elevation of blood pressure, n (%)				
I–II	5 (20.0)	0 (0.0)		
III–IV	0 (0.0)	0 (0.0)	5.135	0.023

## Data Availability

The data generated in the present study may be requested from the corresponding author.
